# Nomogram based on inflammatory indices for differentiating intrahepatic cholangiocarcinoma from hepatocellular carcinoma

**DOI:** 10.1002/cam4.2823

**Published:** 2020-01-05

**Authors:** Lang Chen, Furong Zeng, Lei Yao, Tongdi Fang, Mengting Liao, Jing Long, Liang Xiao, Guangtong Deng

**Affiliations:** ^1^ Xiangya Hospital Central South University Changsha China

**Keywords:** aspartate transaminase‐to‐neutrophil ratio index (ANRI), differential diagnosis, hepatocellular carcinoma (HCC), intrahepatic cholangiocarcinoma (ICC), nomogram

## Abstract

**Objective:**

To establish nomogram based on inflammatory indices for differentiating intrahepatic cholangiocarcinoma (ICC) from hepatocellular carcinoma (HCC).

**Methods:**

A cohort of 422 patients with HCC or ICC hospitalized at Xiangya Hospital between January 2014 and December 2018 was included in the study. Univariate and multivariate analysis was performed to identify the independent differential factors. Through combining these independent differential factors, a nomogram was established for differential diagnosis between ICC and HCC. The accuracy of nomogram was evaluated by using receiver operating characteristic (ROC) curve, calibration curve, and decision curve analysis (DCA). The results were validated using a prospective study on 98 consecutive patients operated on from January 2019 to November 2019 at the same institution.

**Results:**

Sex (OR = 9.001, 95% CI: 3.268‐24.792, *P* < .001), hepatitis (OR = 0.323, 95% CI: 0.121‐0.860, *P* = .024), alpha‐fetoprotein (AFP) (OR = 0.997, 95% CI: 0.995‐1.000, *P* = .046), carbohydrate antigen 19‐9 (CA199) (OR = 1.016, 95% CI: 1.007‐1.025, *P* < .001), and aspartate transaminase‐to‐neutrophil ratio index (ANRI) (OR = 0.904, 95% CI: 0.843‐0.969, *P* = .004) were the independent differential factors for ICC. Nomogram was established with well‐fitted calibration curves through incorporating these 5 factors. Comparing model 1 including gender, hepatitis, AFP, and CA199 (C index = 0.903, 95% CI: 0.849‐0.957) and model 2 enrolling AFP and CA199 (C index = 0.850, 95% CI: 0.791‐0.908), the nomogram showed a better discrimination between ICC and HCC, with a C index of 0.920 (95% CI, 0.872‐0.968). The results were consistent in the validation cohort. DCA also confirmed the conclusion.

**Conclusion:**

A nomogram was established for the differential diagnosis between ICC and HCC preoperatively, and better therapeutic choice would be made if it was applied in clinical practice.

## Graphical abstract

Nomogram based on inflammatory indices was established for the differential diagnosis between ICC and HCC preoperatively, and better therapeutic choice would be made if it was applied in clinical practice.

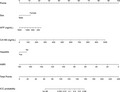

## 1 INTRODUCTION

Primary liver cancer is the sixth most common cancer and the fourth leading cause of cancer‐related mortality worldwide, which consists of hepatocellular carcinoma (HCC), intrahepatic cholangiocarcinoma (ICC) and mixed hepatocellular‐cholangiocarcinoma carcinoma.^1^ Though originating from different cell origins, HCC and ICC frequently share several common etiological risk factors and clinical manifestations, which is a challenge in differential diagnosis between HCC and ICC.^2^, ^3^, ^4^ Because HCC and ICC differ in therapeutic strategies and prognosis, preoperative accurate differentiation and early diagnosis are necessary to improve the treatment outcome.^5^, ^6^, ^7^ However, in current clinical practice, the gold standard of differential diagnosis still depends on the pathological examination after liver resection.^8^ Therefore, an accurate preoperative differentiation is of great significance in clinical decision‐making.

Many efforts on preoperative differential diagnosis have been made in recent years. Magnetic resonance imaging (MRI) and contrast‐enhanced computerized tomography (CT) are most applied to discriminate the two subtypes, but hardly to differentiate small ICC from HCC in cirrhotic livers due to their common enhancement patterns.^9^, ^10^, ^11^, ^12^, ^13^, ^14^ Of contrast‐enhanced ultrasound (US), the risk of misdiagnosis of ICC for HCC is also not negligible.^15^, ^16^ Besides, alpha‐fetoprotein (AFP) and carbohydrate antigen 19‐9 (CA199) are regarded as optimal serum biomarkers to distinguish HCC from ICC while the diagnostic sensitivity and specificity of these biomarkers are unsatisfactory.^7^, ^17^, ^18^ Thus, better preoperative prediction models are needed to differentiate HCC from ICC.

Serum inflammatory indices are reflective of the systematic inflammation, which play an essential role in cancer development and progression.^19^ Inflammatory indices have shown to be prognostic in primary liver cancer including the neutrophil‐to‐lymphocyte ratio (NLR), platelet‐to‐lymphocyte ratio (PLR), lymphocyte‐to‐monocyte ratio (LMR), aspartate aminotransferase‐to‐platelet ratio index (APRI) and aspartate aminotransferase‐to‐neutrophil ratio index (ANRI).^20^, ^21^, ^22^, ^23^, ^24^, ^25^ What is more, inflammatory indices were used to differentiate the existence of microvascular invasion in HCC.^26^ Whether inflammatory indices could be used to distinguish HCC from ICC has never been explored. The objective of our study was to develop a nomogram based on inflammatory indices for the preoperative differential diagnosis between ICC and HCC.

## 2 METHODS

### 2.1 Patients

With the approval of the Xiangya Hospital of Central South University, a retrospective study was conducted on a training cohort of HCC and ICC patients who underwent partial hepatectomy between January 2014 and December 2018. The study was conducted in compliance with the Declaration of Helsinki and written informed consent was obtained from all patients for their data to be used for research. Patients did not receive financial compensation. The inclusion criteria were as follows: (a) patients were above 18 years old; (b) underwent surgical resection; (c) pathological diagnosis of HCC and ICC. Diagnostic criteria were based on Guidelines for Diagnosis and Treatment of Primary Liver Cancer in China (2017 Edition); (d) imaging data and serum inflammatory data were available before surgery. The exclusion criteria were as follows: (a) patients were less than 18 years old; (b) patients were diagnosed as metastatic tumor before; (c) patients had infection before surgery; (d) imaging data and serum inflammatory data were incomplete or unavailable. From January 2019 to November 2019, using the same inclusion and exclusion criteria, an independent cohort of consecutive HCC and ICC patients who underwent partial hepatectomy was prospectively studied. These patients constituted the validation cohort of the study.

### 2.2 Clinicopathologic variables

Patients’ demographic variables were obtained including age, sex, body mass index, history of diabetes, hypertension, and hepatitis. Number of tumor nodules, tumor size and ascites were included in patients’ imaging data based on contrast‐enhanced MRI and contrast‐enhanced CT. Serum examination included indocyanine green retention rate at 15 minutes (ICG‐R15), AFP, carcinoembryonic antigen (CEA), CA199, albumin (ALB), total bilirubin (TBIL), direct bilirubin (DBIL), alanine transaminase (ALT), aspartate transaminase (AST), alkaline phosphatase (ALP), prothrombin time (PT), international normalized ratio (INR), neutrophil, lymphocyte, monocyte, platelet, hemoglobin, NLR, PLR, LMR, APRI, and ANRI. In our study, NLR was measured by the neutrophils count divided by the lymphocytes count; PLR was measured by the platelet count divided by the lymphocyte count; LMR was measured by the lymphocytes count divided by the monocytes count. APRI was obtained using the following formula: APRI = [AST level (/ULN)/Platelet counts (10^9^/L)] × 100. ANRI was calculated by AST divided by the neutrophils count.

### 2.3 Statistical analysis

Continuous variables were expressed as mean ± SD and Student's *t* test was used for the comparison. Categorical variables were expressed as frequency and compared using Fisher exact test. Multivariate logistic regression analysis was used to identify the independent ICC differential factors. Nomogram was plotted based on these independent differential factors. Receiver operating characteristic (ROC) curve analysis was used for comparison between our nomogram and other models based on the concordance index (C index). A calibration curve with 422 bootstrap samples was employed to measure the accuracy of the nomogram. For the external validation of the nomogram, the established nomogram was used to calculate the total points of each patient in the validation cohort, and ROC curve and calibration curve were plotted to assess the accuracy of the nomogram. The decision curve analysis (DCA) was conducted to evaluate the clinical utility of the nomogram and other models through quantifying net benefits against a range of threshold probabilities. SPSS 22.0 (SPSS Inc, Chicago, IL, USA), EmpowerStats, State SE and R 3.1.2 software (Institute for Statistics and Mathematics) were performed in our analysis. *P* < .05 was considered statistically significant.

## 3 RESULTS

### 3.1 Clinicopathologic Characteristics of Patients

During the study period, 520 consecutive patients who met the inclusion criteria were enrolled, and divided into training cohort and validation cohort. In the training cohort, a total of 422 primary live cancer patients were enrolled into this study, including 375 HCC patients and 47 ICC patients. For the validation cohort, 98 consecutive patients were studied, consisting of 88 HCC patients and 10 ICC patients. The clinicopathologic characteristics of the patients are listed in Table 1. The baseline clinicopathologic data were comparable between the training and validation cohorts.

**Table 1:** Characteristics of patients in hepatocellular carcinoma (HCC) and intrahepatic cholangiocarcinoma (ICC)

Variables	Training (n = 422)	Validation (n = 98)	*P* value
Demographics and history			
Age (years)	52.71 ± 11.37	54.17 ± 12.66	.264
Sex			
Man	358	84	.826
Woman	64	14	
BMI	23.03 ± 3.20	23.40 ± 2.88	.323
Diabetes			
No	392	87	.173
Yes	30	11	
Hypertension			
No	343	84	.302
Yes	79	14	
Etiology			
Hepatitis	341	79	.965
Others	81	19	
Tumor type			
HCC	375	88	.790
ICC	47	10	
Preoperative blood tests			
ICG‐R15 (%)	5.97 ± 6.22	8.34 ± 8.97	.055
AFP (ng/mL)	288.47 ± 428.09	326.08 ± 381.44	.4251
CEA (ng/mL)	3.42 ± 8.86	5.84 ± 37.52	.526
CA199 (ng/mL)	50.87 ± 129.87	33.55 ± 103.53	.2181
ALB (g/L)	41.07 ± 4.53	40.54 ± 4.43	.300
TBIL (μmol/L)	15.02 ± 13.78	13.52 ± 6.78	.295
DBIL (μmol/L)	6.92 ± 8.18	7.02 ± 3.58	.905
ALT (U/L)	44.01 ± 40.57	45.89 ± 64.17	.782
AST (U/L)	49.64 ± 45.12	45.96 ± 37.17	.453
PT (s)	13.88 ± 5.89	13.78 ± 1.55	.862
INR	1.07 ± 0.10	1.08 ± 0.13	.4241
Neutrophil (10^9^/L)	3.64 ± 2.36	3.38 ± 1.49	.293
Lymphocyte (10^9^/L)	1.53 ± 0.97	1.39 ± 0.55	.159
Monocyte (10^9^/L)	0.81 ± 1.67	0.73 ± 0.72	.650
Platelet (10^9^/L)	163.76 ± 76.05	161.35 ± 80.96	.7801
HB (g/L)	142.21 ± 67.00	138.68 ± 16.53	.606
NLR	2.76 ± 2.19	2.83 ± 2.21	.758
PLR	121.97 ± 73.45	50.94 ± 21.24	<.001
LMR	3.34 ± 1.79	3.21 ± 1.33	.482
APRI	1.17 ± 5.34	0.91 ± 1.06	.626
ANRI	16.74 ± 17.69	15.04 ± 10.16	.361
Preoperative imaging			
Tumor number			
Solitary	362	79	.199
Multiple	60	19	
Tumor size (cm)	6.42 ± 4.61	6.30 ± 3.85	.818
Ascites			
No	411	94	.432
Yes	11	4	

Note

Categorical variables are expressed as frequency. Continuous variables are expressed as mean ± standard deviation.

Abbreviations: AFP, α‐fetoprotein level; ALB, albumin; ALT, alanine transaminase; ANRI, AST‐to‐neutrophil ratio index; APRI, AST‐to‐platelet ratio index; AST, aspartate transaminase; CA199, cancer antigen 199; CEA, carcinoembryonic antigen; DBIL, direct bilirubin; ICG‐R15, indocyanine green retention rate at 15 min; INR, international normalized ratio; LMR, lymphocyte‐to‐monocyte ratio; NLR, neutrophil‐to‐lymphocyte ratio; PLR, platelet‐to‐lymphocyte ratio; PT, prothrombin time; PTA, prothrombin activity; TBIL, total bilirubin.

### 3.2 Univariate and multivariate analysis of differential factors between ICC and HCC

**Figure 1:**
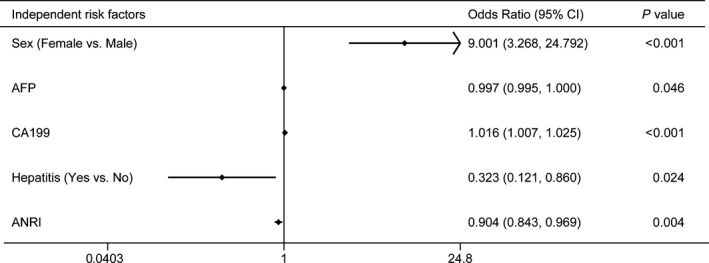
Plot of independent differential factors between intrahepatic cholangiocarcinoma (ICC) and hepatocellular carcinoma (HCC) based on multivariate logistic regression analysis

In the training cohort, the univariate analysis suggested that age (*P* = .033), sex (*P* < .001), hepatitis (*P* < .001), AFP (*P* < .001), CA199 (*P* < .001), INR (*P* < .001), Neutrophil (*P* < .001), Platelet (*P* < .001), NLR (*P* = .018), PLR (*P* < .001), LMR (*P* = .023), and ANRI (*P* < .001) were potential differential factors between ICC and HCC (Table 2). Subsequently, all these potential differential factors were recruited into multivariate logistic analysis. Only sex (OR = 9.001, 95% CI: 3.268 ‐ 24.792, *P* < .001), hepatitis (OR = 0.323, 95% CI: 0.121‐0.860, *P* = .024), AFP (OR = 0.997, 95% CI: 0.995‐1.000, *P* = .046), CA199 (OR = 1.016, 95% CI: 1.007‐1.025, *P* < .001) and ANRI (OR = 0.904, 95% CI: 0.843‐0.969, *P* = .004) were the independent differential factors for the presence of ICC (Figure 1).

**Table 2:** Characteristics of patients in hepatocellular carcinoma (HCC) and intrahepatic cholangiocarcinoma (ICC)

Variables	HCC (n = 375)	ICC (n = 47)	*P* value
Demographics and history			
Age (years)	52.29 ± 11.44	56.04 ± 10.35	**.033**
Sex			
Man	331	27	**<.001**
Woman	44	20	
BMI	23.03 ± 3.22	23.04 ± 3.08	.985
Diabetes			
No	347	45	.558
Yes	28	2	
Hypertension			
No	304	39	.845
Yes	71	8	
Etiology			
Hepatitis	327	14	**<.001**
Others	48	33	
Preoperative blood tests			
ICG‐R15 (%)	6.16 ± 6.52	4.69 ± 3.49	.213
AFP (ng/mL)	320.05 ± 439.77	36.46 ± 178.37	**<.001**
CEA (ng/mL)	2.79 ± 4.19	8.34 ± 23.20	.108
CA199 (ng/mL)	26.79 ± 28.16	242.98 ± 324.79	**<.001**
ALB (g/L)	40.99 ± 4.53	41.71 ± 4.58	.310
TBIL (μmol/L)	14.62 ± 10.64	18.25 ± 28.36	.389
DBIL (μmol/L)	6.78 ± 7.26	8.03 ± 13.55	.538
ALT (U/L)	44.01 ± 40.55	44.02 ± 41.19	.999
AST (U/L)	50.68 ± 46.70	41.31 ± 28.67	.180
PT (s)	13.99 ± 6.23	13.01 ± 0.84	.284
INR	1.07 ± 0.10	1.02 ± 0.07	**<.001**
Neutrophil (10^9^/L)	3.47 ± 2.32	5.01 ± 2.29	**<.001**
Lymphocyte (10^9^/L)	1.54 ± 1.01	1.46 ± 0.62	.588
Monocyte (10^9^/L)	0.82 ± 1.74	0.75 ± 1.01	.791
Platelet (10^9^/L)	156.90 ± 74.21	218.55 ± 68.55	**<.001**
HB (g/L)	143.37 ± 70.76	132.98 ± 17.00	.371
NLR	2.59 ± 1.70	4.13 ± 4.25	**.018**
PLR	116.07 ± 70.77	169.00 ± 78.17	**<.001**
LMR	3.41 ± 1.82	2.78 ± 1.47	**.023**
APRI	1.26 ± 5.66	0.51 ± 0.35	.356
ANRI	17.68 ± 18.41	9.24 ± 6.53	**<.001**
Preoperative imaging			
Tumor number			
Solitary	320	42	.657
Multiple	55	5	
Tumor size (cm)	6.35 ± 4.79	7.00 ± 2.78	.364
Ascites			
No	346	47	.620
Yes	11	0	

Note

Categorical variables are expressed as frequency. Continuous variables are expressed as mean ± standard deviation.

ICG‐R15, indocyanine green retention rate at 15 min.

Abbreviations: AFP, α‐fetoprotein level; ALB, albumin; ALT, alanine transaminase; ANRI, AST‐to‐neutrophil ratio index; APRI, AST‐to‐platelet ratio index; AST, aspartate transaminase; CA199, cancer antigen 199; CEA, carcinoembryonic antigen; DBIL, direct bilirubin; INR, international normalized ratio; LMR, lymphocyte‐to‐monocyte ratio; NLR, neutrophil‐to‐lymphocyte ratio; PLR, platelet‐to‐lymphocyte ratio; PT, prothrombin time; PTA, prothrombin activity; TBIL, total bilirubin.

Bold indicates statistically significant values (*P* < .05).

### 3.3 Development and validation of a nomogram for ICC differential diagnosis

**Figure 2:**
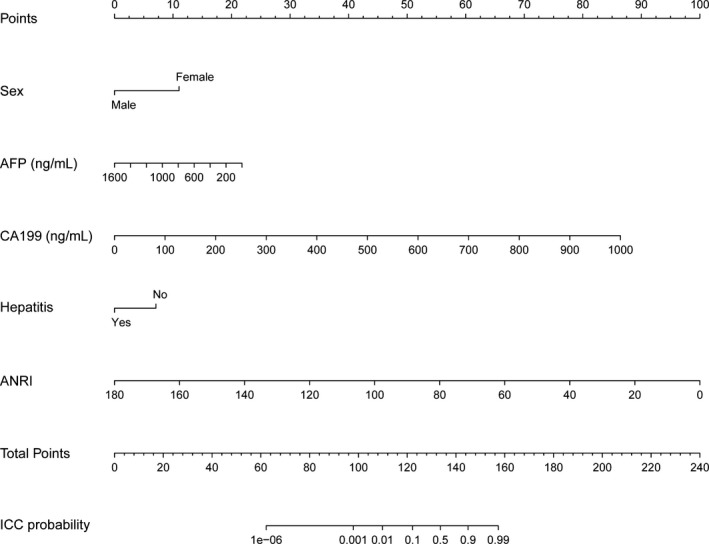
Nomogram for differentiating ICC and HCC

**Figure 3:**
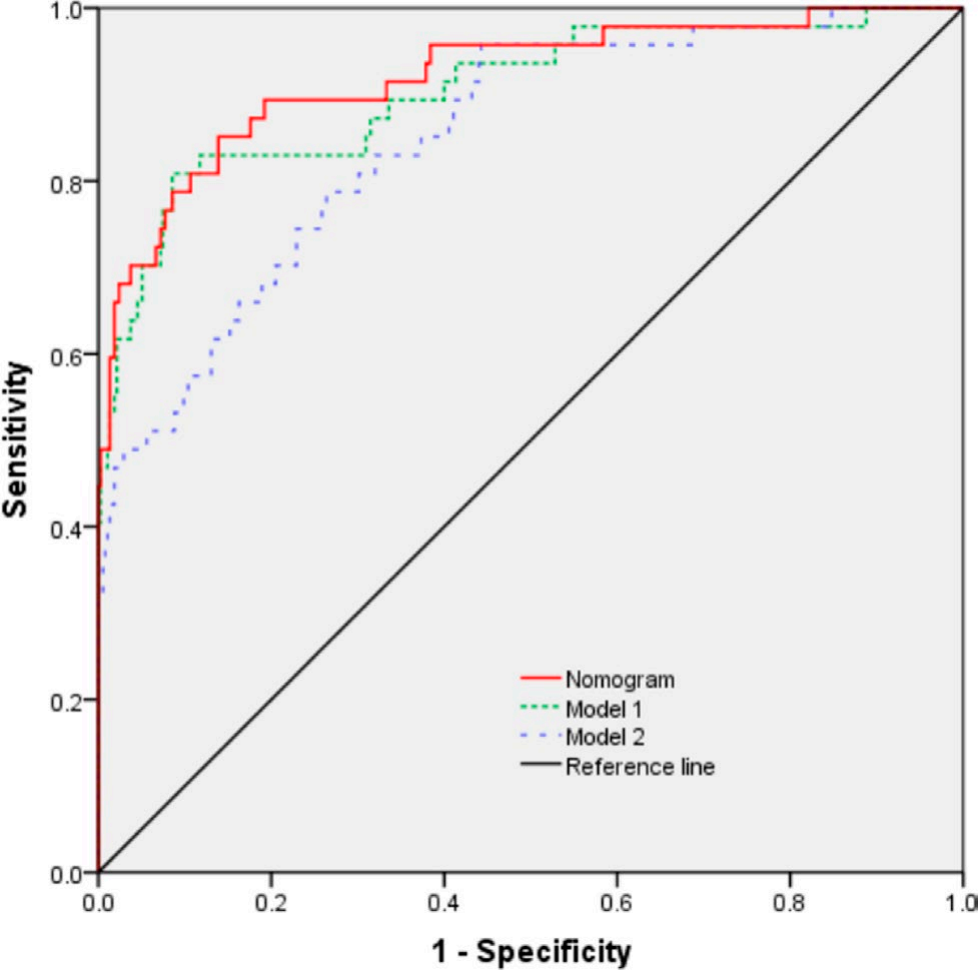
Receiver operating characteristic (ROC) curve of the nomogram and other models in the training cohort. Model 1 consists of sex, hepatitis, alpha‐fetoprotein (AFP), and carbohydrate antigen 19‐9 (CA199); model 2 includes AFP and CA199

**Figure 4:**
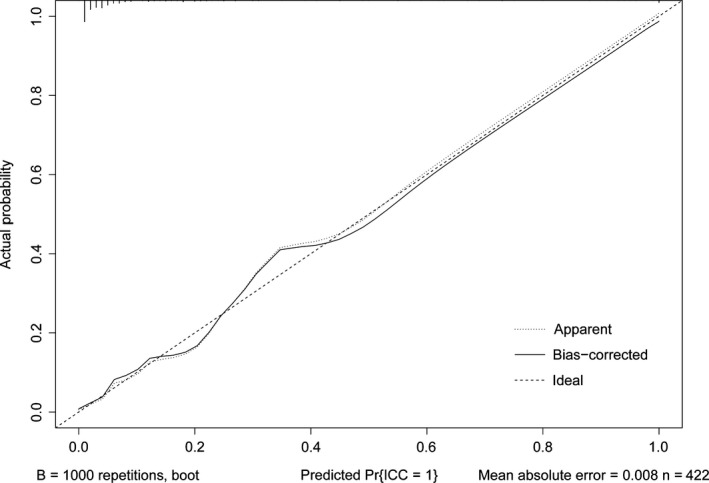
Calibration curve of the nomogram in the training cohort

**Figure 5:**
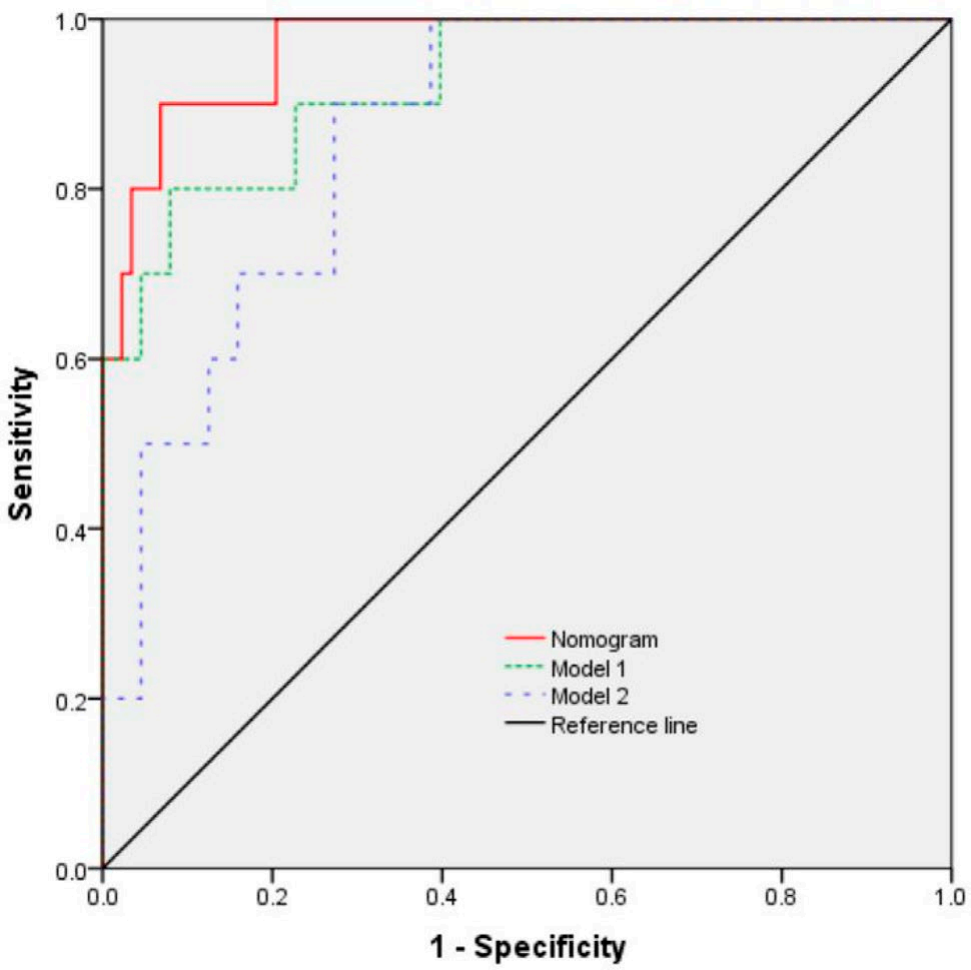
Receiver operating characteristic (ROC) curve of the nomogram and other models in the validation cohort

**Figure 6:**
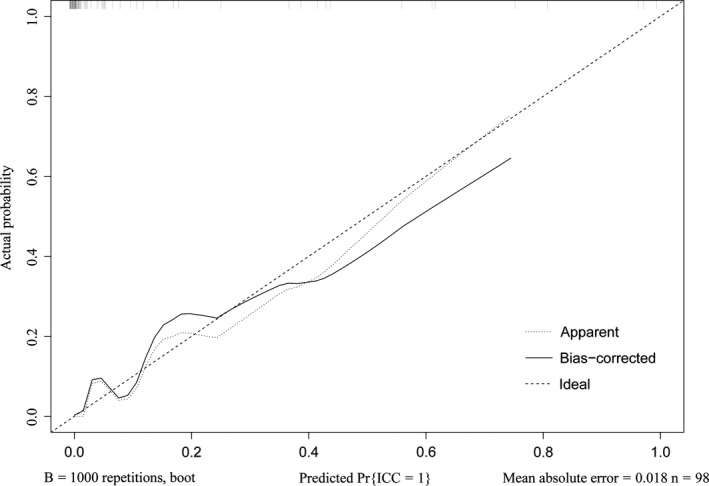
Calibration curve of the nomogram in the validation cohort

**Figure 7:**
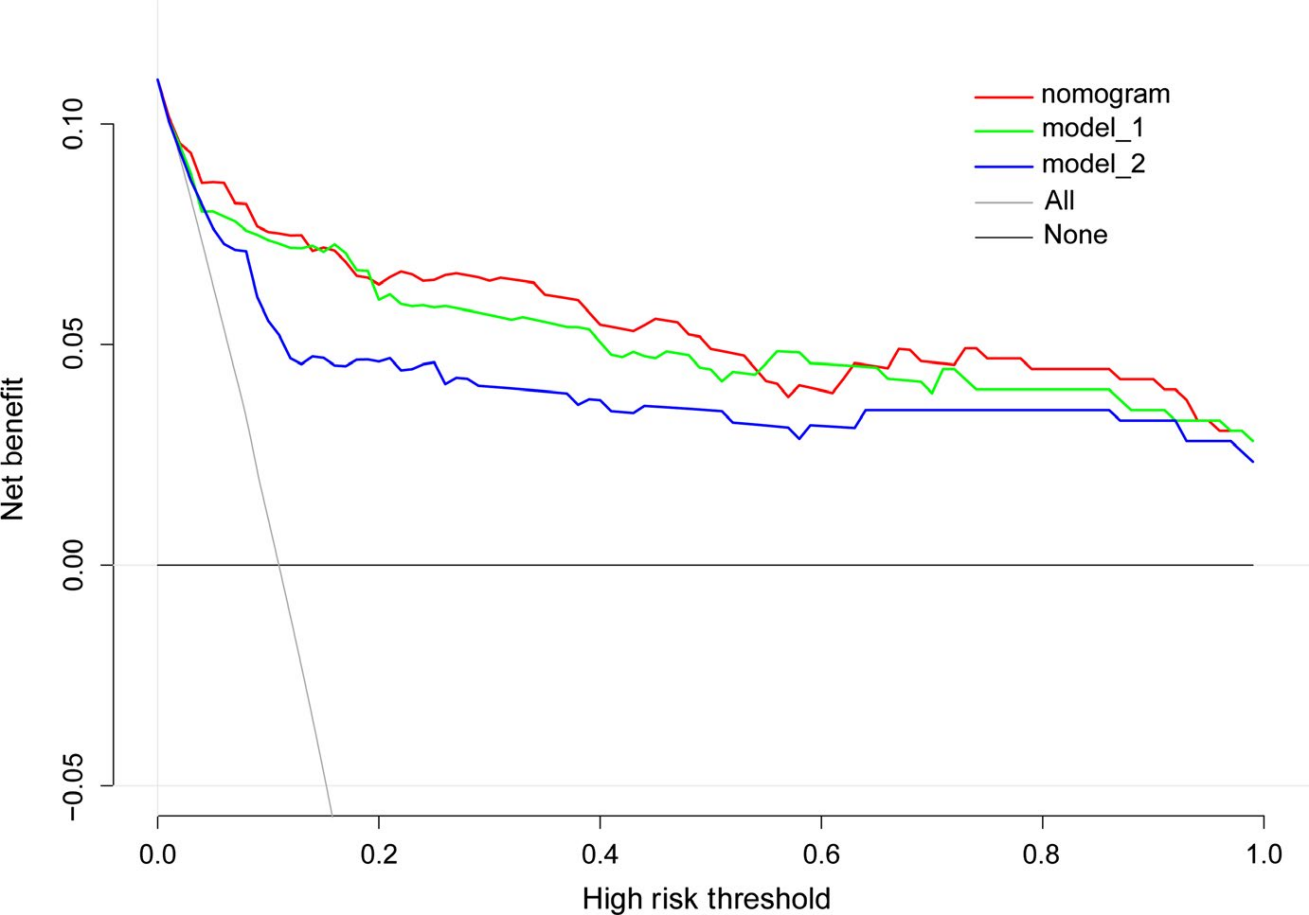
Decision curve analysis of our nomogram and other models

The independent differential factors between ICC and HCC were further employed to establish an ICC risk estimation nomogram (Figure 2). To highlight the significance of ANRI, we built model 1 including gender, hepatitis, AFP, and CA199. Also, we established model 2 enrolling AFP and CA199, which was mostly used in clinic practice. Comparing with model 1 (C index = 0.903, 95% CI: 0.849‐0.957) and model 2 (C index = 0.850, 95% CI: 0.791‐0.908), the nomogram showed a better discrimination for ICC and HCC with an C index of 0.920 (95% CI, 0.872‐0.968) (Figure 3). The calibration curves revealed sufficient agreement between the nomogram and actual histopathologic confirmation on surgical specimens (Figure 4). In the validation cohort, comparing with model 1 (C index = 0.925, 95% CI: 0.842‐1.000) and model 2 (C index = 0.865, 95% CI: 0.772‐0.957), the nomogram displayed a C index of 0.967 (95% CI, 0.925‐1.000) for the differentiation of ICC risk (Figure 5). There was also a good calibration curve for the ICC risk estimation (Figure 6). DCA has been used to assess the clinical value of models which integrates the preferences of patients into the analysis.^27^, ^28^ DCA showed that using this nomogram to distinguish ICC from HCC added more benefit compared with model 1 and 2 (Figure 7).

### 3.4 Risk of ICC based on the nomogram scores

Sensitivity and specificity for the ICC differential nomogram at different predicted probabilities were summarized in Table 3. Based on the maximum of the Youden index, the optimal cutoff for the nomogram predicted probability were set to be 0.088. The sensitivity, specificity, positive predictive value, and negative predictive value when used in differentiating ICC from HCC were 85.11%, 86.13%, 43.48%, and 97.88%, respectively (Table 4).

**Table 3:** Differential efficacy of the nomogram at different predicted probability

Predicted probability	Sensitivity	Specificity	PPV	NPV
0.01	97.87%	32.53%	15.38%	99.19%
0.05	89.36%	74.93%	30.88%	98.25%
0.10	80.85%	86.40%	42.70%	97.30%
0.20	70.21%	93.60%	57.89%	96.16%
0.30	65.96%	97.60%	77.50%	95.81%
0.40	61.70%	98.13%	80.56%	95.33%

Abbreviations: NPV, negative predictive value; PPV, positive predictive value.

**Table 4:** Differential efficacy of the nomogram at optimal predicted probability

Variables	Value
Sensitivity	85.11%
Specificity	86.13%
Positive predictive value	43.48%
Negative predictive value	97.88%
Positive likelihood ratio	6.14
Negative likelihood ratio	0.17
ROC area (95%CI)	0.92 (0.87‐0.97)
Predicted probability	0.088

Abbreviations: CI, confidence intervals; ROC, receiver operating characteristic.

## 4 DISCUSSION

Many models have been put forward to distinguish ICC from HCC based on MRI, CT, and US, but their value of clinical use is limited due to the lack of costly high‐resolution equipment and experienced radiologists especially in some developing areas.^5^, ^10^, ^11^, ^12^, ^13^, ^16^ Besides, considering that many high‐risk patients are ineligible for the application of MRI and CT, we aim to establish a simple but accurate differential diagnosis model for differentiating ICC from HCC for clinical use.

In our study, we found that sex, hepatitis, AFP, CA199, and ANRI were the independent differential factors between ICC and HCC through the multivariable logistic regression analysis. Based on these independent differential factors, we established a nomogram to distinguish ICC from HCC. Hepatitis, AFP, and ANRI were negatively related to ICC, while female and CA199 were positive factors in this ICC differential nomogram. Hepatitis is the main risk factor of HCC and AFP is secreted by about half of the HCC tumor.^29^, ^30^ Though AFP was not recommended for the HCC diagnosis by the “American Association for the Study of Liver Disease” (AASLD) and the “European Association for Study of the Liver” (EASL), AFP is still a part of the diagnostic criteria of HCC in Asian countries.^31^, ^32^ Epidemiological studies have shown a higher incidence of HCC in men than in women. A possible reason is that higher adiponectin in women could activate AMPKα and p38α which confers protection against HCC.^33^ Admittedly, hepatitis, AFP, sex, and CA199 have been demonstrated to distinguish ICC from HCC in many studies which are consistent with our conclusion, while lower ANRI in ICC than in HCC has never been investigated.^7^, ^17^, ^18^ In our case, AST in ICC patients is lower than in HCC patients because HCC patients usually are infected with hepatitis virus which was correlated with the AST level.^34^ Furthermore, more neutrophils are recruited by ICC cells through expressing chemokine ligand 5.^35^ ANRI, a ratio of AST divided by neutrophils count, amplifies the effects of AST and neutrophil in differential diagnosis between ICC and HCC, which could reflect the hepatocyte injury and the tumor burden and progression.^20^ That may also explain why ANRI not other inflammatory indices could be used to differentiate ICC from HCC.

In current clinical practice, clinicians usually employ AFP and CA199 to distinguish ICC and HCC.^7^, ^17^, ^18^ Compared to our nomogram (C‐index = 0.920), this model (model 2) has a lower C‐index value of 0.850. To highlight the significance of ANRI, we established model 2 which consists of sex, hepatitis, AFP, and CA199. Compared to model 1 (C‐index = 0.903), our nomogram performs well in differentiating ICC from HCC. Thereafter we used the consecutive patients from our hospital to test the accuracy of our model. The nomogram was validated by the C‐index value of 0.967 in validation cohorts. Conventionally, nomogram is evaluated using sensitivity, specificity, and C‐index which failed to assess the clinical value. DCA is a well‐established method to assess the benefits of a diagnostic test across a range of patient preference for accepting risk of undertreatment and overtreatment to facilitate decisions about test selection and use.^27^, ^28^ In our case, more benefits were added using our nomogram than other models, suggesting that using our nomogram to differentiate ICC from HCC would be the best decision for all patients, regardless of individual values, and a clinician can use this approach uniformly.

Our nomogram is helpful in the differential diagnosis between ICC and HCC preoperatively, which can guide on therapeutic treatment. For example, HCC patients have multiple curative intent options while surgical resection is the only curative therapy for ICC patients.^36^ Unresectable HCC patients could receive liver transplantation while the indication of liver transplantation for ICC remains less defined because of concerns of poor prognosis.^37^, ^38^ With our nomogram, we can pick up the HCC patients who were misdiagnosed as ICC before. These patients could regain the chance of liver transplantation or other curative treatments. Also, our nomogram may serve as a selection tool during randomized clinical trials on neoadjuvant treatment for recruiting ICC patients.

To our knowledge, this is the first nomogram based on inflammatory indices to differentiate ICC from HCC. We highlight the importance of ANRI in the differential diagnosis between ICC and HCC. However, our study has some limitations. First, this analysis was a retrospective study based on data from a single hospital. Second, an external validation is necessary to confirm the differential value of the nomogram. Finally, due to analysis based on clinicopathologic and serum data, specific markers such as PTTG and microRNA‐204, might further improve the accuracy of the nomogram.^39^, ^40^, ^41^, ^42^, ^43^

In conclusion, we demonstrated that sex, hepatitis, AFP, CA199, and ANRI are the independent differential factors between ICC and HCC. By combining these easily accessible differential factors, a differential diagnostic nomogram was established for optimal discrimination between ICC and HCC. The nomogram could optimally differentiate ICC from HCC preoperatively and help with therapeutic options and prognosis evaluation.

## Data Availability

The data that support the findings of this study are available on request from the corresponding author. The data are not publicly available due to privacy or ethical restrictions.
